# The realities of adolescent sexual behaviours in Nigeria: a narrative review

**DOI:** 10.4314/ahs.v24i2.30

**Published:** 2024-06

**Authors:** David Bamidele Olawade, Akinsola J Asaolu, Yusuff Adebayo Adebisi, Fiyinfoluwa T Asaolu, Aderonke Odetayo, Aanuoluwapo Clement David-Olawade

**Affiliations:** 1 Department of Allied and Public Health, School of Health, Sport and Bioscience, University of East London, London, United Kingdom; 2 Department of Community Medicine, Faculty of Public Health, University of Ibadan, Oyo State, Nigeria; 3 Centre for Research and Development, Kingsway Hospital, Derbyshire Healthcare NHS Foundation Trust, Derby, United Kingdom; 4 Faculty of Pharmacy, University of Ibadan, Oyo State, Nigeria; 5 Faculty of Health and Life Sciences, De Montfort University, United Kingdom; 6 School of Nursing, Tung Wah College, Hong Kong SAR, China; 7 Department of Nursing, University of Derby, Derby, United Kingdom

**Keywords:** Sexual health, sexual behaviour, adolescent, young people, Nigeria

## Abstract

**Background:**

Adolescence is a critical period of development during which young people experience significant physical, cognitive, and social changes. Adolescent sexual behaviors can have significant consequences for their physical and mental health, as well as for their social and economic well-being. In Nigeria, the majority of adolescents have their sexual debut before the age of 18, and many do not have access to comprehensive sexuality education or sexual and reproductive health services.

**Objective:**

We conducted a narrative review to discuss how early adolescence, in conjunction with a variety of social and environmental variables, influences adolescents' risky sexual behaviours in Nigeria.

**Methods:**

A narrative review was conducted to explore the realities of adolescent sexual behaviors in Nigeria. Searches were conducted in PubMed, Google Scholar, Medline, and PubMed Central using predetermined search terms. The articles were reviewed and analyzed and then the findings were discussed narratively.

**Results:**

Various factors, including sexual maturation, peer association, and environment play key roles in an adolescents' drive toward a first sexual experience. Many adolescents participate in risky sexual activities that may impact their health and well-being. These risky sexual behaviours, such as early sexual debut, lack of or improper use of condoms, multiple sexual partners, put them at high risk of contracting sexually transmitted infections (STIs), as well as unintended adolescent pregnancy.

**Conclusion:**

Teenagers are momentarily endangered with various risky sexual behaviors as those who lack parental sex education are at greater risk. Hence, parent-child communication should be encouraged to curtail risky sexual habits among teenagers.

## Introduction

According to the United Nations, teenagers are defined as those between the ages of 10 and 19, and they account for roughly 16.0% (1.2 billion) of the world population^1^. In Sub-Saharan Africa (SSA), adolescents account for approximately 23.0% of the region's population^1^. Accord ing to the United Nations Population Fund, teenagers comprised 22.1% (28 million) of Nigeria's population in 2010 and have since risen to 45.7% (45.7 million) of the country's overall population^2^. Adolescence brings about both physical and mental changes. This includes the period preceding the start of puberty, which is characterised by an increase in secondary features, and the post-puberty phase. At this point, the care-free days of childhood begin to ebb away, replaced with an abrupt awareness of maturity and the necessity to accept responsibility for one's actions. This presents them with a number of obstacles that they must overcome in order to develop into better adults^3^,^4^. The obstacles they face may overwhelm them as they attempt to adjust to their new status^4^. As a result, teenagers experience a variety of vicissitudes, including worry, shocks, discoveries, delight, fear of the unknown, and independence^4^. It can be perplexing and contradictory for a teenager at times since they are attempting to comprehend the fast changes occurring within themselves and may find it challenging to comprehend and manage these changes^3^. Parents have a critical role in the lives of teenagers and are also tasked with the responsibility of nurturing and guiding them through this stage of life^5^.

Adolescence is characterized by increasing interest in the opposite sex and experimenting with a variety of risk-taking behaviours^5^. Adolescents at this time have transitioned from an innocent phase in which they can get away with their appearance and worry less about gender differences to a phase in which they are obsessed with physical and sexual beauty. From this point on, they develop a casual friendship that may develop into a romantic one^6^. Sexual maturity, on the other hand, typically arrives before physical and emotional maturity and is followed by acts and judgments that teenagers must make based on their sexual lives without regard for the hazards connected with it. As a result, teenagers frequently attain sexual maturity prior to achieving mental and emotional development. Adolescents develop at varying rates; girls often reach physical maturity earlier than boys. This disparity frequently leads to girls developing romantic attraction prior to boys and so initiating sexual behaviours early^7^.

According to the United Nations^1^, there is an early age of sexual behaviour initiation throughout adolescence. Adolescence's developmental and behavioural changes might raise one's chance of getting STIs and having an unplanned pregnancy. Late adolescence (15 to 19 years) is crucial since this is when sexual introduction and experimentation frequently begin^8^. The purpose of this narrative review is to understand how early adolescence, in conjunction with a variety of social and environmental variables, influences adolescents' hazardous sexual behaviours in Nigeria. The objectives are as follows:

- To provide an overview of teenagers' hazardous sexual behaviours in Nigeria.
- To ascertain the causes and variables that contribute to teenagers engaging in hazardous sexual behaviours.
- To gain a better understanding of how parental communication affects teenage sexual behaviours.

## Method

We conducted a non-systematic short narrative review to answer the aim of the study and the methods used in this paper following the guideline by Green et al.^9^. Searches were conducted in PubMed, Google Scholar, Medline, and PubMed Central using the following search terms: “Early adolescence”, “Sexual Behaviours”, “Sexual Maturity”, “Sexual Health”, “Hazardous Health Behaviour”, “Teenagers”, “Young People”, “Nigeria”. Only cross-sectional studies published in English Language up to July 2022 were included. The exclusion criterion was any other data sources that do not provide information regarding the topic. Three members of the research team were involved in the literature review to gather data for this study independently and in case of duplication of references or disagreement, a consensus was reached through discussions. The authors also utilized a snowball approach i.e., we reviewed relevant articles extracted from reference lists of the articles identified in the literature search. The extracted data were discussed narratively to answer the aim and objectives of the study. The narrative review was conducted to synthesize existing literature on adolescent sexual behaviors in Nigeria, provide an overview of the factors that influence such behaviors, and identify the challenges that adolescents face and the implications for public health and policy. The narrative review aims to provide a comprehensive understanding of the current state of knowledge on adolescent sexual behaviors in Nigeria, and to inform public health and policy efforts to improve the sexual and reproductive health of adolescents in the country.

### Practices of Risky sexual behaviours among adolescents

Sexual risky behaviour is frequently characterised as behaviour that enhances an individual's risk of getting sexually transmitted diseases such as HIV/AIDS or adolescent pregnancy^5^. Consequences of hazardous sexual behaviour, such as illness, adolescent pregnancy, or childbirth, can be detrimental to overall family health, as well as mother and child health. Children delivered to young mothers are susceptible to low birth weight, stillbirth, or delivery problems such as vesicovaginal fistula, all of which contribute to infant mortality^10^,^11^.

In Nigeria, 3.0% of 15–19-year-olds are HIV positive and have unwanted pregnancies^12^. According to the National Demographic and Health Survey^13^. approximately 4.7 million teenagers in Nigeria were sexually active at the time of the survey and had sex within the previous three months. About 45% of these teenagers did not utilise any kind of contraception^14^,^15^. Numerous sexually active teenagers engage in hazardous sexual behaviours as well^16^,^17^. In unmarried teenagers, risky sexual behaviour includes early sexual beginning, unprotected sexual intercourse without or inconsistent condom usage, many sexual partners, and having sex while under the influence of inhibition-lowering substances such as psychotropic drugs and alcohol^5^,^18^. According to the National Youth Risk Behaviour Survey, 47.0% of high school students in the United States are sexually active^19^. Furthermore, a regional survey estimated that between 24 and 75.0% of teenage men had intercourse prior to marriage in Asia, 44.0% to 66.0% in Latin America, 45.0% to 75.0% in Sub-Saharan Africa, and 80.0% in certain affluent nations^20^. Sexual behaviour among adolescents in sub-Saharan Africa and Nigeria is undergoing change^21^,^22^. Similarly, a study revealed that nearly half of teenagers (47.4%) were sexually active and participated in different types of hazardous sexual behaviour^23^. Additionally, another study revealed that 50.0% of secondary school students aged 15-19 years were sexually active in their research^24^. Moharson-Bello et al. and Adeomi et al. both observed a lower prevalence in their studies performed in the country's south-western region^25^,^26^. They reported that around 22.9% and 28.3% of respondents, respectively, had prior sexual exposure^25^,^26^. Recent research from four institutions in southern Nigeria discovered that hazardous sexual behaviours among teenage undergraduate students are based on the quality of early adolescent sexual education provided by parents^27^. As a result, adolescents who were not exposed to excellent sex education beginning in early puberty had a higher prevalence of numerous sexual partners and unsafe intercourse^27^.

These studies demonstrate that a sizable proportion of teenagers are sexually active and engage in a variety of hazardous sexual behaviours. Sexual activity is prevalent among both sexes in Nigeria and around the world, according to studies. According to NARHS, one-quarter of teenage boys and half of the adolescent females in Nigeria are sexually active^28^. NDHS also claimed that about half (46.2%) of girls had had intercourse, compared to 22% of males^13^. In contrast, several other researchers have found that a larger proportion of males had engaged in sexual activity^25^,^29^.

### Early sexual initiation and associated factors

Early sexual start lengthens the time span during which an unintended pregnancy and other sexually transmitted diseases are possible^22^,^30^,^31^. Sexually active adolescents are more likely to have numerous sexual partners and are less likely to use condoms during sexual intercourse^5^. Sexual activity among teenagers is high, and the age of first sexual encounter is decreasing, according to two different studies^32^,^33^. In the United States, the 2013 National Youth Behaviour Survey showed that 6.0% of adolescents had their first sexual encounter before reaching the age of 13^19^. A study of female adolescents in Kenya discovered that the average age at first sex was 16.2 years, that 54.0% of them had sexual experience by the age of 16, and that 84.0% of them had their first sexual experience by the age of 18^34^. Surprisingly, sexual activity began relatively early for four of the females in the research, with their first sexual experience occurring at the age of eight^34^. In Nigeria, a similar trend is evident, as an increasing number of teenagers initiate sexual contact at an early age. A survey of 350 in-school teenagers in Anambra State discovered that 34.4% were already sexually active, and 68.3% had their first sexual encounter between the ages of 13 and 16^35^. Similarly, another study observed that almost a quarter of Plateau State's secondary school students were sexually active by the age of 13^29^. Similarly, research conducted in Cross River State discovered that the average age of sexual debut was 13.7 years^36^.

According to the 2008 NDHS study in Nigeria, the mean age of first sexual intercourse was 16.7 years, with 15.0% of females and 6.0% of males reporting having sex^37^. A similar pattern was observed in the NDHS 2013 data, with 24% having sexual intercourse by age 15 and 54.0% by age 18^38^. On the other hand, additional research indicates that some adolescents engage in sexual activity in their late teens, with the average age of sexual debut being 17 years^13^,^19^,^28^.

Early sexual initiation is a significant public health concern and is associated with negative health outcomes such as unintended pregnancies, STIs, and mental health issues^22^,^30^,^31^. Factors associated with early sexual initiation include socio-economic status, family factors, peer pressure, lack of parental supervision, early onset of puberty, substance use and lack of access to sexual education^28^,^34^. Addressing these factors through interventions such as family-based programs, school-based sexual education and access to healthcare and reproductive services can help to reduce the rate of early sexual initiation and improve the sexual and reproductive health outcomes of adolescents.

### Unprotected sexual intercourse

When condoms are worn regularly and appropriately, sexual intercourse greatly decreases the risk of infection for both parties^39^. The advantages of condom use exceed the dangers of not using them; they also serve as a contraceptive^39^. Adolescents frequently engage in sexual activity without using contraception, which results in a greater rate of unintended pregnancy, unsafe abortions, HIV/AIDS, and other sexually transmitted infections (STIs) among young adults^28^,^40^. The prevalence of hazardous sexual behaviour among teenagers, which includes inconsistent condom usage, has steadily increased. According to studies, less than 20% of Nigerian teenagers used a condom during their first intercourse^39^. Adolescents are particularly at risk for engaging in unprotected sexual intercourse due to a lack of knowledge about sexual and reproductive health, peer pressure, and societal attitudes and cultural norms that may discourage the use of condoms or other forms of contraception. To address this, there is a need for comprehensive sexuality education that includes information about the importance of using condoms and other forms of contraception, as well as easy access to condoms and other forms of contraception. Additionally, efforts should be made to address societal attitudes and cultural norms that may discourage the use of condoms or other forms of contraception.

### Multiple sexual partners

Adolescents frequently maintain many sexual partners as a result of their uncontrollable sexual desires at this stage of their lives^40^. Having multiple sexual partners increases the number of people one is exposed to sexually, which increases the chances of coming into contact with someone who has an STI or unintended pregnancies. In a Tanzanian research, teenagers reported having between one and seven sexual partners, with around 15% of sexually active adolescents having numerous sexual relationships^41^.

In Bida, Niger State, Northern Nigeria, a survey found that half of the sexually active teenagers had intercourse with their boyfriend or girlfriend, 7.5% with their fiancé or fiancée, and 3.6% with an older male or female partner (sugar daddy; sugar mummy)^42^. Around 1.3% have engaged in sexual activity^42^. Additionally, a study conducted in Rivers State, South-eastern Nigeria, discovered that almost half of respondents had more than one sexual partner, with around 6.0% having more than five sexual partners^43^. Similarly, around 54% of teenagers in Niger State had more than one sexual relationship^44^. Additionally, 22.6% of sexually active teenagers in Cross River State had more than one sexual relationship, while about one-third of sexually active adolescents in Delta State had more than one sexual partner^36^,^45^. According to Moharson-Bello et al., the proportions of individuals who had numerous relationships and single partners were nearly equal at 43.9% and 42.7%, respectively^25^. Numerous repercussions of hazardous sexual behaviour, such as sexually transmitted illnesses, adolescent pregnancy, and childbirth, can be detrimental to overall family health, mother and child health, and child health. Children delivered to young mothers are susceptible to low birth weight, stillbirth, or delivery problems such as vesicovaginal fistula, all of which contribute to mortality^11^,^23^.

### Reasons and factors associated with adolescent risky sexual behaviours

While teenagers' motives for participating in premarital sexual activities vary, they are typically motivated by curiosity, peer pressure, pleasure, financial gain, or natural desires^32^. Additionally, some of them participate in sexual behaviour for the sake of enjoyment, closeness, and companionship^36^. In Anambra State, 50.0% of teenagers engaged in sexual activities as a result of peer pressure, 27.5% for financial gain, 16.7% for personal fulfilment, 4.2% out of curiosity, and 1.7% owing to a lack of parental and relative direction^35^. In Niger State, 58.0% of residents engaged in sexual activity for enjoyment, 22.0% for fertility testing, and 7.0% to improve sexual aptitude^44^. According to research conducted in Abia State, 5.4% of females were drugged, 4.1% were raped, 7.4% were compelled, and 14.2% were misled, while 23.0% did it out of curiosity and 4.1% out of biological urges^46^. Additionally, research performed among female teenagers in Mushin, Lagos State, discovered that the primary motivation for sexual involvement was curiosity; uncontrollable natural urges and alcohol also played a role^47^. This, however, has been demonstrated in numerous studies to be a primary reason for teenagers engaging in sexual behaviours through curiosity, peer influence, enjoyment, and a lack of parental direction.

Adolescents from impoverished and financially challenged families are more likely than those from financially secure families to engage in sexual activity at a young age^42^,^48^,^49^. Economic hardship, poverty, and the perception of money and material gain are all variables that contribute to girls engaging in early sex^50^. In co-educational schools, peer pressure and socioeconomic status may also have an unfavourable effect on teenagers' sexual behaviour^51^. Female genital mutilation (FGM), which is done in a number of African tribes with the hope of reducing female child promiscuity, has been related to having numerous sexual partners^52^. Healthy family communication is a critical element in determining how teenagers behave sexually^53^. Communication between adolescents and their parents, as well as parent receptivity to sexual conversations, are critical in postponing early sexual debut, particularly in females^54^,^55^. Adolescents' sexual habits and behaviour are also influenced by the structure and operation of their families^33^. A study of teenagers' sexual behaviour in Nigeria discovered that those raised in polygamous households engaged in more early sexual activity than those raised in monogamous households^29^. Additionally, teenagers' sense of connection to their family reduces their chance of engaging in hazardous sexual behaviours^29^. Adolescents' haphazard media exposure has also been discovered as a strong predictor of early sexual initiation; viewing foreign and domestic films, as well as pornography, has been identified as a facilitator of premarital sex^32^,^47^,^56^. However, religion is a significant impediment to premarital sex, since it is one of the primary reasons some teenagers refrain from sex^50^.

Adolescent risky sexual behaviours are influenced by a complex interplay of factors such as lack of knowledge about sexual and reproductive health, peer pressure, societal attitudes and cultural norms, substance abuse, economic factors, mental health, and family factors^42^,^48^,^49^,^50^. Adolescents who lack access to accurate information about sexual and reproductive health may engage in risky behaviours such as unprotected sexual intercourse and multiple sexual partners. Peer pressure can also push adolescents to engage in risky behaviours. Societal attitudes and cultural norms that discourage the use of condoms or other forms of contraception or that encourage multiple sexual partners can also increase the likelihood of risky behaviours. Substance abuse, economic factors, mental health issues and family factors can also contribute to the likelihood of engaging in risky sexual behaviours. Addressing these factors can help to reduce the prevalence of risky sexual behaviours among adolescents and to improve their sexual and reproductive health outcomes^50^.

### Influence of parent-child communication on adolescent sexual behaviours

Little research has been conducted on the relationship between family communication and hazardous sexual behaviour among teenagers^57^. As other writers have highlighted, the causes of these behaviours are not just personal, but also structural and societal^30^,^31^,^58^. Parents, being the most continuous influence in their children's lives, are well-positioned to impact adolescents' health, personal development, and transition to sexual life^59^. When the parent-child relationship is authoritarian and lacks open communication, it can have a detrimental effect on both teenagers' self-esteem and the choices they make about health-related behaviours and lifestyles^55^. Furthermore, numerous research in poor countries have indicated that parent-child communication on sexual and reproductive health issues is unusual^60^-^62^. Wherever it occurs, parents' instructions about sexuality are frequently unclear, for example, ‘do not play with boys^63^.

Despite the fact that India's policies and programmes recognise the importance of actively engaging parents and adolescents in navigating safe and healthy adulthood transitions, evidence about parent-child interaction on sensitive topics such as risky sexual behaviours and unhealthy lifestyles is scant^64^. Parent-child communication, particularly on sensitive personal topics, and sex discussions are severely restricted by cultural taboos^64^,^65^. Parenting requires effective communication betweenparent and kid^66^. There is evidence that parent-child dialogue regarding sex-related issues is uncommon. It is frequently stressful and uncomfortable, particularly interactions with dads in sub-Saharan Africa^60^. According to Markham et al., regular, and open parent-child communication is predicted to reduce the likelihood of sexual risk-taking in adolescents by fostering more responsible teenage behaviour^67^. The parent-child bond is shattered as a result of this lack of communication, and home becomes a weird world to the learner, while school appears to be the other. Increased parental participation in assisting teenagers to make appropriate lifestyle and sexual behaviour choices has been suggested in SSA nations^53^,^68^,^69^. However, literature on parent-child communication is scarce in low- and middle-income countries (LMICs) such as Nigeria^55^,^66^. Open parent-child communication is critical for the family's health and growth^53^. It has been demonstrated to have a protective effect on teenagers' psychosocial development and behaviour^70^. However, studies have shown that parents who communicate openly and frequently with their children have children who are less likely to engage in substance use and misuse^71^,^72^. Additionally, the severity of family communication difficulties increases the likelihood of adolescents developing behavioural disorders^73^. Adolescents who identify with their parents' views on sexual behaviour and lifestyle concerns are less likely to participate in risky behaviours in sub-Saharan Africa^65^,^74^.

Parent-child communication plays a critical role in shaping adolescent sexual behaviors^59^. Research has shown that adolescents who have open and effective communication with their parents about sexual and reproductive health are more likely to engage in healthier sexual behaviors and to delay the onset of sexual activity^53^,^70^. Parents who provide accurate and age-appropriate information about sexual and reproductive health, and who model healthy communication and relationships, can help their children to make informed choices about their sexual behaviors. Additionally, parents who actively listen to their children and create an open, non-judgmental environment can help their children to feel more comfortable discussing sensitive topics like sex. On the other hand, lack of communication or communication that is overly restrictive or that shames or stigmatizes sexuality can lead to increased risk-taking behaviors among adolescents.

## Recommendation

Based on the findings of the narrative review, several recommendations can be made to address the realities of risky adolescent sexual behaviours in Nigeria:

1. Increase access to comprehensive sexuality education: Adolescents need accurate and age-appropriate information about sexuality and sexual and reproductive health in order to make informed choices. This education should be provided in schools and other community settings, and should be inclusive of all adolescents, regardless of gender, sexual orientation, or socioeconomic status.
2. Improve access to sexual and reproductive health services: Adolescents should have access to a wide range of sexual and reproductive health services, including family planning, STI testing and treatment, and prenatal care. These services should be provided in a confidential, youth-friendly, and non-judgmental manner.
3. Address societal attitudes and cultural norms: Societal attitudes and cultural norms can influence adolescent sexual behaviours. To address this, there is a need for community-based interventions that aim to change attitudes and norms related to adolescent sexuality and sexual and reproductive health.
4. Address poverty and lack of access to education: Adolescents from low-income families and those who lack access to education are at higher risk of engaging in risky sexual behaviours. To address this, there is a need for policies and programs that aim to reduce poverty and increase access to education for all adolescents.
5. Address gender-based discrimination: Adolescent girls are at higher risk of experiencing gender-based discrimination, which can negatively impact their sexual and reproductive health. To address this, there is a need for policies and programs that aim to promote gender equality and empower adolescent girls.

It is important to note that these recommendations should be implemented in a way that is inclusive and sensitive to the cultural context and local realities of Nigeria, and that they should involve the active participation of adolescents and the communities in which they live.

## Conclusion

Numerous teenagers, particularly those between the ages of 15 and 18, are at risk of sexually transmitted diseases and unwanted pregnancy as a result of multiple sexual partners, a lack of or ineffective use of condoms, and an early sexual debut. Adolescents reared in a setting devoid of parental sex education in early adolescence have a significant proclivity for many sexual partners and unsafe sex, especially after they reach university. Additionally, peer pressure, insatiable curiosity, financial need, and a lack of parental direction all contribute to teenagers engaging in hazardous sexual behaviours. The importance of parent-child communication cannot be overstated since it shapes adolescent behaviour from an early age. As these teenagers mature, parents must foster positive contact with them and teach them about puberty and sex development in order to minimise the occurrence of dangerous sexual behaviours.
